# Microscopy‐Detected Enteric Parasite‐Compatible Stages and Arthropod Contaminants in Ready‐to‐Eat Salads in Sylhet City Corporation, Bangladesh

**DOI:** 10.1155/japr/8294177

**Published:** 2026-11-06

**Authors:** Umama Lubna, S. M. Sertaz Islam, Bijoy Banik, Kazi Mehetazul Islam, Saiful Islam

**Affiliations:** ^1^ Department of Livestock Production and Management Sylhet Agricultural University Sylhet Bangladesh sau.ac.bd; ^2^ Department of Parasitology Sylhet Agricultural University Sylhet Bangladesh sau.ac.bd; ^3^ Department of Epidemiology and Public Health Sylhet Agricultural University Sylhet Bangladesh sau.ac.bd

**Keywords:** arthropod contaminants, enteric parasites, microscopy, ready-to-eat salad, Sylhet

## Abstract

Parasitic stages can exist in ready‐to‐eat salads. This cross‐sectional study was aimed at examining the ready‐to‐eat salad samples from 10 commercial salad areas of Sylhet City Corporation, Sylhet city between October 2025 and April 2026. A total of 100 samples were obtained from street food vendors, mid‐range restaurants and fine dining restaurants. Diagnosis was made on direct wet mount method, examination of iodine‐stained cells and modified formalin‐ether sedimentation, respectively. Overall, 26 samples (26.0%; 95% CI: 17.7%–35.7%) contained at least one classifiable finding. Enteric parasite compatible stages were found in 14 (14.0%) samples, which include five *Balantioides coli* cysts, three *E. histolytica/dispar/moshkovskii*‐complex, one *Giardia* sp. cyst, one trematode egg, and one *Ascaris* sp. stage. No cases of nonenteric arthropod contamination (mites) were detected in any of the 13 samples (13%; 95% CI: 7.1%–21.2%) recorded separately. The results indicated that RTE salads can have microscopic contaminants, though they did not document the viability or pathogenicity or the risk of infection. A detailed morphological identification and adequate number of fine‐dining subgroups should be taken into consideration for further investigation.

## 1. Introduction

Contamination of fresh produce with foodborne pathogens represents an important food‐safety concern, as contaminated fruits and vegetables have been implicated in numerous foodborne disease outbreaks [1]. A Global Burden of Disease 2021 analysis estimated an age‐standardized disability‐adjusted life‐year (DALY) rate of 1020.15 per 100,000 population for enteric infections, with a higher burden observed in regions with lower sociodemographic indices [2]. Ready‐to‐eat salads take a particularly high‐risk niche in this topography because they are unheated, variation in the steps involved in handling the food between farm and customer, and potentially allow the survival of pathogenic organisms [3].

In Bangladesh, there is a lack of systematic data on parasitic contamination of salads and fresh produce, but it always remained alarming. A study conducted in Mymensingh, Bangladesh, reported parasitic contamination in 61.5% (123/200) of raw vegetable samples, with *Ascaris lumbricoides* and *Strongyloides stercoralis* among the most frequently detected parasites [4]. Other studies in Bangladesh have also documented fecal‐indicator contamination in household food and environmental samples, as well as microbiological quality at an unsatisfactory level in some street‐vended foods in Dhaka [5, 6]. In the local market of Dhaka, 29.6% of raw salad vegetable samples brought at least one parasitic species, with coriander being the most contaminated sample and vendor hygiene practices being a statistically significant predictor [7]. Studies of raw vegetables have documented protozoan cysts and/or helminth eggs, demonstrating the diversity of parasitic stages reported across different settings [8–10].

The most frequently isolated protozoa in contaminated produce include *Balantidium coli, Entamoeba histolytica*, as well as *Cryptosporidium* spp., most of which are known to carry a risk of zoonotic transmission [11]. Studies in Asia and Africa have reported trematode eggs and soil‐transmitted helminths, including *A. lumbricoides*, hookworm species, *S. stercoralis*, and *Trichuris trichiura*, on raw vegetables. Irrigation with untreated or insufficiently treated wastewater can contribute to contamination of crops with helminth eggs and protozoan cysts [12, 13]. During microscopic examination, mites were also observed and recorded separately as nonenteric arthropod contaminants to document extraneous biological material in salad samples. However, their presence does not establish contamination with intestinal parasites.

The characteristics of street food vending in developing countries include low access to clean water, lack of food hygiene training, lack of personal hygiene practices, and being in unregulated outdoor locations [14, 15]. A national representative study of Bangladesh found that no food handler in the street food sector paid attention to washing hands with soap at any observed food‐handling event, such as preparing salads, and that soap was made available at handwashing stations accessible to the staff at only 33% of the formal restaurants [16]. Sylhet City Corporation is a growing urban municipality in northeastern Bangladesh, covering approximately 79.5 km^2^ across 42 wards [17]. Its commercial areas include street‐food vendors, mid‐range restaurants, and fine‐dining establishments where ready‐to‐eat salads are served for immediate consumption. Although studies in Bangladesh have documented parasitic contamination of raw vegetables sold in markets [4, 7], evidence for restaurant‐served salads in Sylhet remains limited. Unlike market samples, salads collected at the point of service have already undergone preparation and handling, which may influence contamination [18]. Therefore, this study is aimed at documenting the frequency and morphological diversity of enteric parasite‐compatible stages and nonenteric arthropod contaminants in ready‐to‐eat salads collected from the commercial areas of Sylhet City Corporation, Bangladesh.

## 2. Materials and Method

### 2.1. Study Design and Setting

A cross‐sectional study was conducted to establish the prevalence of parasitic contamination in ready‐to‐eat salads sold by food sellers in the Sylhet City Corporation, Bangladesh, from the period October 2025 to April 2026. The sampling was evenly done over 10 business locations of the city namely: Zindabazar, Bondor Bazar, Baruthkhana, Mirabazar, Tilagor, Nayasarak, Sobhanighat, Mirboxtula, Chowhatta, and Kumarpara.

### 2.2. Sample Collection

A total of 100 ready‐to‐eat salad samples were collected from 10 selected commercial areas of Sylhet City Corporation. Within each area, food establishments were randomly selected for sample collection. No formal sample‐size calculation was performed. Samples were collected in sterile containers and transported within 2 h of collection at ambient temperature and processed within 4 h of arrival in laboratory. The collected samples were placed into three different categories namely: street food vendors, mid‐range restaurants, and fine‐dining establishments. Only six fine‐dining samples were obtained during the study period, resulting in limited representation of this category.

### 2.3. Parasitological Examination

All samples underwent standardized parasitological examination. The salad samples were processed without prior measurement of their individual weights. During sample preparation, each salad sample was initially rinsed under running tap water to remove loosely attached surface debris before being immersed into 1000 mL of normal saline solution (0.9% sodium chloride) and gently agitated. To distinguish the suspended components, the washing solution was centrifuged at 447 × g for 15 min, or 2000 rpm, with a rotation radius of 10 cm. The supernatant was carefully removed, and the sediment was collected using a dropper. A drop of sediment was placed on a clean glass slide, a cover slip was placed over it, and the slide was examined under a light microscope using 10× and 40× objectives. The whole procedure followed the direct wet mount method with standard protocols [19]. For accurate identification of eggs, cysts, and larvae, a drop of the sediment was mixed with a drop of Lugol′s iodine solution and examined as in a simple smear under the light microscope. Moreover, samples were further assessed by the modified formalin ether sedimentation method to detect parasites [20]. Different types of parasites were identified according to their morphological features, and the prevalence of these parasites was recorded for the type of vegetable and fruit sample examined. Microscopic structures lacking sufficient diagnostic morphological features for reliable identification were not classified as parasitic stages and were excluded.

### 2.4. Statistical Analysis

The salad samples were the unit of analysis. Microscopic findings were categorized as enteric parasite‐compatible stages or nonenteric arthropod contaminants. The numbers of individual microscopic detections and distinct positive samples were reported separately. Sample‐level prevalence was calculated using the total number of examined samples as the denominator, with exact (Clopper–Pearson) 95% confidence intervals. The distributions of establishment categories, salad types, and ingredients were summarized descriptively. Inferential subgroup comparisons were not reported for the revised outcomes. Quantitative public health risk index was not estimated in this study, and detection of mites was summarized separately from enteric parasite‐compatible findings. Confidence intervals assume independent samples; clustering by establishment and area was not accounted for.

## 3. Results and Discussion

### 3.1. Overall Prevalence

Of the 100 salad samples, 14 (14.0%; 95% CI: 7.9%–22.4%) contained enteric parasite‐compatible stages, and 13 (13.0%; 95% CI: 7.1%–21.2%) contained nonenteric mites. Overall, 26 samples (26.0%; 95% CI: 17.7%–35.7%) contained at least one classifiable finding. The 26 positive samples yielded 28 detections (15 enteric, 13 mites). The two groups often overlapped in one sample, so their sample counts should not be summed. Detection of a particular stage does not establish the viability, pathogenicity, or infection to consumer.

The prevalence of enteric parasite compatible stages was lower than the values reported for raw vegetables in Mymensingh (61.5%) [4] and Dhaka (29.6%) [7]. Several factors may contribute in this case such as examination of raw produce whereas ready‐to‐eat salads may have undergone preparation and handling; tap‐water prerinse may have removed loosely attached stages; and only a small volume of sediment examination per sample. Pooled estimates for parasitic contamination of fresh produce also vary widely across regions and methods [21, 22].

### 3.2. Sample Distribution by Establishment Tier and Salad Type

Of the 100 salad samples, 73 were obtained from street food vendors, 21 from mid‐range restaurants, and 6 from fine‐dining establishments. Eighty‐nine samples were mixed salads and 11 were single‐ingredient salads (Table 1). The substantially smaller fine‐dining group limits the precision of subgroup estimates. Because the previously reported establishment‐ and salad‐type positivity findings combined enteric stages, mites, and unclassified structures, they are not presented as enteric parasite‐specific prevalence estimates in the revised analysis.

**Table 1:** Distribution of examined salad samples by establishment category and salad type (*N* = 100).

Variable	Category	Examined (*n*)	% of samples
Establishment tier	Street food	73	73.0
	Mid‐range	21	21.0
	Fine dining	6	6.0
Salad type	Mixed	89	89.0
	Single	11	11.0
**Total**	Overall	100	100.0

*Note:* Values in this table describe the composition of the sample; they are not contamination prevalence estimates.

### 3.3. Microscopic Findings Recovered

A total of 28 morphologically classifiable microscopic detections were recorded in 26 of the 100 examined salad samples (Table 2). Enteric parasite‐compatible stages accounted for 15 detections (53.6%) involving 14 samples (14.0%), whereas mites accounted for 13 detections (46.4%) involving 13 samples (13.0%). The enteric findings included *B. coli* cysts (*n* = 5), cysts morphologically compatible with the *E. histolytica/dispar/moshkovskii* complex (*n* = 3), unsporulated protozoan cyst‐like structures (*n* = 2), unspecified protozoan cyst‐like structures (*n* = 2), a trematode egg (*n* = 1), a *Giardia* spp. cyst (*n* = 1), and an *Ascaris* spp. form (*n* = 1). The occurrence of multiple protozoan and helminth‐compatible stages in fresh produce has been documented worldwide, supporting the diversity of parasitic contaminants that may be encountered in raw vegetables [11, 22]. Nonenteric arthropod contaminants included 12 unspecified mites and one *Demodex* mite. Mites were reported separately and were not considered evidence of intestinal parasitic contamination.

**Table 2:** Enteric parasite‐compatible stages and nonenteric arthropods detected in ready‐to‐eat salads (*N* = 100).

Group	Parasitic stage	*n*	% of samples (*N* = 100)	% of detections (*n* = 28)
Enteric parasite‐compatible	*Balantidium coli* (cyst)	5	5.0	17.9
	^∗^ *Entamoeba* spp.	3	3.0	10.7
	Unsporulated protozoan cyst‐like structure	2	2.0	7.1
	Unspecified protozoan cyst‐like structure	2	2.0	7.1
	Trematode egg	1	1.0	3.6
	*Giardia* spp. (cyst)	1	1.0	3.6
	*Ascaris* spp.	1	1.0	3.6
	Subtotal, enteric	15	14.0	53.6
Nonenteric arthropods	Mite (unspecified)	12	12.0	42.9
	*Demodex mite*	1	1.0	3.6
	Subtotal, mites	13	13.0	46.4
Total		28	26.0	100.0

*Note:* *n* denotes individual detections. The 15 enteric parasite‐compatible detections occurred in 14 distinct samples, and 13 mites occurred in 13 samples. In total, 28 classifiable detections were distributed across 26 distinct samples; the categories overlap in one sample. Percentages of samples use distinct samples as numerators and *N* = 100 as the denominator. Detection percentages use 28 as the denominator. Morphologically unclassifiable findings were excluded.

^∗^ *Entamoeba* spp. = *Entamoeba histolytica*/*dispar*/*moshkovskii* complex (cyst).

The recovered enteric spectrum was dominated by protozoa. *Balantioides coli* cysts were the most frequently identified organism (5.0% of samples). *B. coli* is a ciliate that infects humans and pigs; pigs are the main reservoir, and human infection ranges from asymptomatic to dysenteric disease [23, 24]. Cysts compatible with the *E. histolytica/dispar/moshkovskii* complex were found in 3% of samples. Light microscopy cannot differentiate these species, so species identity and pathogenic potential could not be confirmed without molecular testing [25]. One *Giardia* sp. cyst was detected. Microscopy has limited sensitivity compared with molecular methods, and a cyst does not indicate viability [26]. One *Giardia* sp. cyst was detected. Microscopy has limited sensitivity compared with molecular methods, and a cyst does not indicate viability [27, 28]. Four protozoan structures were recorded only as cyst‐like structures (“unsporulated”, *n* = 2; “unspecified”, *n* = 2) because diagnostic features were insufficient for further identification.

Mites accounted for 13 of the 28 classifiable detections (46.4%) and were kept separate from enteric parasite‐compatible stages. Their recovery indicates the presence of extraneous arthropod material in the sampled salads, not intestinal parasitic infection. One *Demodex*‐like mite was recorded; as procedural blanks were not used, potential sample‐handling and laboratory sources could not be distinguished, and the finding should not be interpreted further.

### 3.4. Descriptive Findings by Establishment Category

Establishment‐specific prevalence estimates for enteric parasite‐compatible findings could not be calculated from the available summary‐level data because the findings were not cross‐tabulated by establishment category. Therefore, inferential comparisons between establishment categories were not performed. Fine‐dining establishments contributed only six of the 100 samples. This small group and the uneven distribution across tiers reduce the precision of establishment‐specific estimates and prevent robust inferences about the relative safety of fine‐dining restaurants and street food vendors. Previous investigations of food‐handling conditions in Bangladesh have reported inadequate handwashing facilities and hygiene practices among some street‐food vendors [14, 16]. Environmental exposure, ingredient sourcing, washing, and handling may affect contamination; however, their contributions were not directly measured in this study. The present descriptive data should therefore not be used to attribute enteric parasite‐specific risk to any establishment tier.

### 3.5. Ingredient‐Level Screening

The ingredients recorded in the examined salads were cucumber (79 samples), coriander leaves (54), carrot (50), chili (48), onion or onion stalk (36), and lemon (13) (Table 3). Because these ingredients were not mutually exclusive, their percentages do not sum to 100%. No ingredient‐specific associations were estimated for the enteric parasite‐compatible outcome. Previous studies have suggested that leafy herbs, including coriander, may retain soil and biological material because of their leaf structure [29, 30], but this potential mechanism was not directly evaluated here.

**Table 3:** Frequency of salad ingredients recorded in the samples examined (*N* = 100).

Ingredient	Present, *n* (%)	Absent, *n* (%)
Coriander	54 (54.0)	46 (46.0)
Cucumber	79 (79.0)	21 (21.0)
Lemon	13 (13.0)	87 (87.0)
Onion	36 (36.0)	64 (64.0)
Carrot	50 (50.0)	50 (50.0)
*Chili*	*48 (48.0)*	*52 (52.0)*

*Note:* Ingredients were recorded as present or absent; an individual salad could contain multiple ingredients. These counts are descriptive and do not represent parasite‐specific associations. Onion includes onion stalk; coriander refers to coriander leaves.

## 4. Limitations

This study was limited to 10 commercial areas of a single city and one sampling period, preventing assessment of temporal and geographic variation. The samples were unevenly distributed across establishment categories, particularly for fine‐dining restaurants (*n* = 6). A formal priori sample‐size calculation was not performed. Individual salad weights were not measured. Although the salads were collected ready to eat, the initial rinsing under running tap water may have dislodged or removed loosely attached parasitic stages, including helminth eggs, larvae, and protozoan cysts. The extent of potential loss during this step was not quantified; therefore, the reported detection frequencies may underestimate the contamination present at the time of collection. Microscopy did not establish species identity for morphologically similar protozoa or the viability of recovered stages, and procedural negative controls were unavailable. In addition, sample‐level cross‐tabulations for the revised, more restrictive detection outcomes were not available in the supplied summary data, so establishment‐ and ingredient‐specific associations could not be recalculated. Accordingly, the revision reports descriptive prevalence rather than quantitative comparisons of contamination risk. The absence of molecular confirmation limited the identification of morphologically uncertain structures, which were consequently excluded from the analysis [27]. Future studies incorporating molecular confirmation and quantitative risk assessment are recommended.

## 5. Conclusion

Of the 100 ready‐to‐eat salad samples examined in Sylhet City Corporation, 14 (14.0%) contained enteric parasite‐compatible microscopic stages and 13 (13.0%) contained nonenteric mites. Microscopy alone cannot establish species, viability, or infection risk. These findings support further studies using standardized sampling, confirmatory testing, and adequate sample sizes within each establishment category.

## Author Contributions

U.L., S.M.S.I., and S.I. act as lead authors for this study. U.L., S.M.S.I., B.B., K.M.I., and S.I. carried out the study design, field experiments, data analysis, and manuscript writing. U.L., S.M.S.I., and S.I. assisted with morphometrical analysis and writing. S.I. revised the paper.

## Funding

No funding was received for this manuscript.

## Conflicts of Interest

The authors declare no conflicts of interest.

## Data Availability

The data that support the findings of this study are available from the corresponding author upon reasonable request.
